# Reconstructed Epidermis Produced with Atopic Dog Keratinocytes Only Exhibit Skin Barrier Defects after the Addition of Proinflammatory and Allergic Cytokines

**DOI:** 10.1016/j.xjidi.2024.100330

**Published:** 2024-11-26

**Authors:** Daniel Combarros, Rahma Brahmi, Emma Musaefendic, Alizée Heit, Jevgenija Kondratjeva, Fabien Moog, Charline Pressanti, Line A. Lecru, Sabine Arbouille, Catherine Laffort, Dominique Goudounèche, Jessie Brun, Michel Simon, Marie-Christine Cadiergues

**Affiliations:** 1 Small Animal Clinic, École Nationale Vétérinaire de Toulouse, University of Toulouse, Toulouse, France; 2 Toulouse Institute for Infectious and Inflammatory Diseases (Infinity), University of Toulouse, INSERM, CNRS, Paul Sabatier Toulouse III University, Toulouse, France; 3 Clinique vétérinaire Hermes-Plage, Marseille, France; 4 Clinique Vet in the Moon, Mont-de-Marsan, France; 5 Clinique vétérinaire Alliance, Bordeaux, France; 6 Centre de Microscopie Electronique Appliquée à la Biologie, University of Toulouse, Toulouse, France

**Keywords:** Atopic dermatitis, Dog, Inflammation, Model, Skin barrier

## Abstract

Our objectives were to explore epidermal barrier defects in dogs with atopic dermatitis and to determine whether the defects are genetically determined or secondary to skin inflammation. First, the expression of filaggrin, corneodesmosin, and claudin1, analyzed using indirect immunofluorescence in skin biopsies collected from 32 healthy and 32 dogs with atopic dermatitis, was weaker in the atopic skin (*P =* .003). Second, primary keratinocytes of atopic dogs and healthy dogs were used to produce 3-dimensional reconstructed canine epidermis. The expression of the same proteins was analyzed using indirect immunofluorescence, immunoblotting, and RT-qPCR, whereas reconstructed canine epidermis morphology was investigated by transmission electron microscopy, and the barrier was investigated by functional assays. Next, inflammatory cytokines (IL-4, IL-13, IL-31, and TNFα) were added to the culture medium. The morphology, protein expression, and barrier function of the reconstructed canine epidermis were similar whether produced with keratinocytes from healthy dogs or dogs with atopy. Addition of inflammatory cytokines impaired the protein expression and epidermal barrier of the 2 types of reconstructed canine epidermis equally. To conclude, the reduced expression of epidermal barrier proteins observed in vivo was not reproduced in vitro unless cytokines were used, suggesting that it is induced by the inflammatory milieu.

## Graphical abstract

**Figure:**
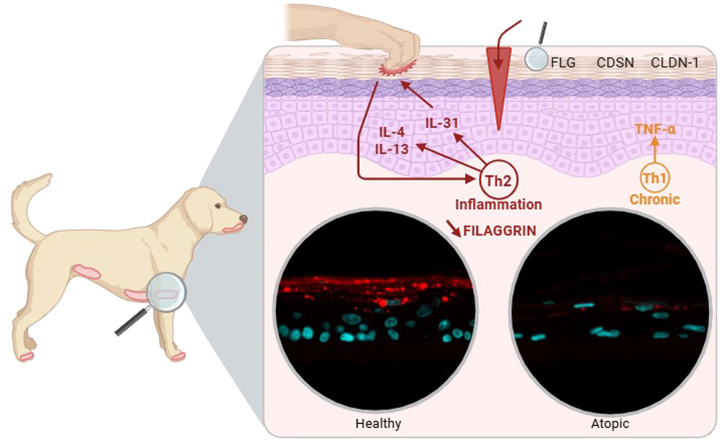

## Introduction

Atopic dermatitis (AD) is a common chronic inflammatory skin disease that affects up to 10% of adult humans and 20% of children in Western countries (Weidinger and Novak, 2016). The pathogenesis of AD is very complex, involving both genetic and environmental factors. On one hand, a defective skin barrier is increasingly considered to be the main trigger of epidermal permeability and percutaneous allergic sensitization (Tsakok et al, 2019). On the other hand, the presence of an abnormal T helper (Th)2/Th22 immune response that is frequently directed toward environmental allergens has been reported (Weidinger and Novak, 2016). The skin barrier defects can be genetically determined (primary), such as the loss-of-function sequence variants of the *FLG* gene that are the major predisposing factor for the development of AD (Palmer et al, 2006). The defects can also be secondary to other factors, including cutaneous Th2 cytokines that have a negative impact on the expression of the epidermal barrier protein (Thyssen and Kezic, 2014).

Although murine models have greatly advanced our understanding of this condition, these models have limitations because mice do not develop the disease spontaneously and do not reproduce all of the phenotypic aspects of the disease (Nakajima et al, 2019). Dogs develop AD spontaneously and share many features of the human condition, with similar prevalence, clinical aspect of the lesions, body areas affected, and genetic predisposition (Hensel et al, 2024), and the 2 species live in a similar environment (Hensel et al, 2015; Marsella and De Benedetto, 2017). Furthermore, recent transcriptome analyses in humans and dogs suffering from AD revealed relatively high molecular concordance (Freudenberg et al, 2019), further supporting the dog as an appropriate model. However, detailed molecular characterization is required to enable confident use of the canine AD model.

The use of in vitro reconstructed 3-dimensional human epidermis is invaluable for understanding the epidermal barrier in both physiological and pathological conditions (Frankart et al, 2012; Huet et al, 2018). For example, reconstruction can advance our understanding of how inflammatory cytokines affect keratinocytes and impair barrier formation (Danso et al, 2014). However, epidermal reconstruction has rarely succeeded using canine keratinocytes.

The main objective of this work was to characterize the expression of epidermal barrier proteins in the skin of AD-affected dogs. The second objective was to investigate whether the skin barrier defects observed in canine AD were primary or secondary to inflammation. To do so, we used an in vitro reconstructed canine epidermis (RCE) model that we developed from primary keratinocytes.

## Results

### The expression of epidermal barrier filaggrin, corneodesmosin, and claudin1 markers was significantly weaker in canine atopic skin

**Figure 1:**
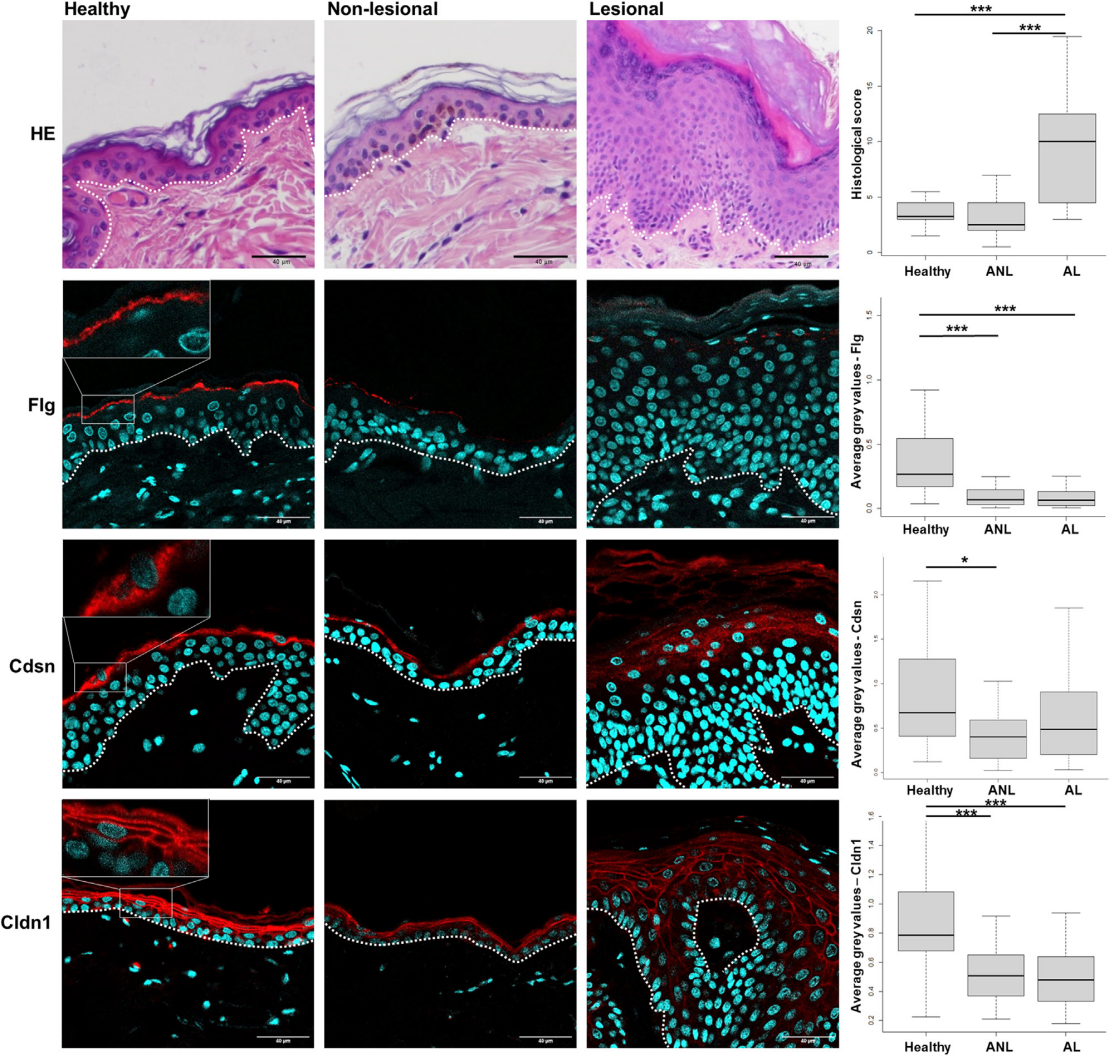
**Reduced FLG, Cdsn, and Cldn1 expression in the skin of dogs with atopy.** Representative images of sections of skin biopsies from healthy dogs and dogs with AD (nonlesional and lesional areas) were stained with H&E and analyzed by indirect immunofluorescence using anti-Flg, -Cdsn, and -Cldn1 antibodies (in red). Nuclei were stained with DAPI (blue). Bar = 40 μm. A histological score depicting atopic skin abnormalities assessed on the H&E-stained samples and quantified gray values of immunodetection signals are plotted on the right. n = 32. Boxplot represent the quartiles, with the central black line being the median. ANL denotes atopic nonlesional, and AL denotes atopic lesional. ∗∗∗*P* < .001 and ∗*P* < .05. AD, atopic dermatitis; CDSN, corneodesmosin.

Thirty-two healthy and 32 dogs with atopy of different breeds were used in this study (Tables 1 and 2). There were no differences between the groups in terms of either median age (3.3 and 3.0 years, respectively) or sex (14 females and 18 males in each group). According to Canine Atopic Dermatitis Extent and Severity Index-4 (Olivry et al, 2014), the animals were suffering from mild (n = 18; 56%), moderate (n = 9; 28%), or severe (n = 5; 16%) AD. Histological evaluation of H&E-stained samples revealed no significant differences between nonlesional atopic skin and healthy controls. As expected, lesional skin showed moderate-to-severe, focal-to-diffuse hyperkeratosis and acanthosis, perivascular-to-interstitial superficial mixed inflammatory infiltrates (lymphocytes, histiocytes, mast cells, plasma cells, and fewer eosinophils), and varying degrees of sebaceous gland hyperplasia. The differences in the histological scores between healthy and lesional atopic skin and between nonlesional and lesional atopic skin were statistically significant (*P* < .001) (Figure 1).

**Table 1:** List of Healthy Dogs Included

Breed	Age, y	Sex	Sample Site
Crossbreed	1	F	Abdomen
Chowchow	1	F	Caudal flank
Beagle	1	F	Abdomen
Beagle	1	F	Abdomen
Ariégeois	1.5	F	Neck
Griffon	1.5	F	Abdomen
Crossed Gascon Saintongeois	2	F	Caudal flank
Crossed Gascon Saintongeois	2	F	Abdomen
Crossed Gascon Saintongeois	2	F	Abdomen
Griffon	2.5	F	Shoulder
Porcelaine	3.5	F	Abdomen
Gascon Saintongeois	4.5	F	Thigh
Labrador	6	F	Abdomen
Terre Neuve	6.5	F	Abdomen
Crossbreed	1	M	Abdomen
Crossed griffon	1	M	Not indicated
Crossed Gascon Saintongeois x griffon	1.5	M	Thorax
Bleu de Gascogne	2.5	M	Abdomen
Gascon Saintongeois	3	M	Thigh
Crossbreed	3	M	Thigh
Jack Russel	3.5	M	Axilla
Crossed griffon	4.5	M	Thigh
Crossed griffon	4.5	M	Thigh
Jadg Terrier	5.5	M	Flank
Griffon	6.5	M	Shoulder
Jack Russel terrier	6.5	M	Abdomen
Crossed griffon	6.5	M	Thigh
Griffon	8	M	Abdomen
Griffon	8	M	Axilla
Griffon	8	M	Thorax
Griffon	8	M	Neck
Fox terrier	9.5	M	Thigh

Abbreviations: F, female; M, male.

**Table 2:** List of Dogs with Atopy Included

Breed	Age, y	Sex	CADESI-04	PVAS	Sample Site	
					Nonlesional	Lesional
Chihuahua	1.5	MS	5.5	5.5	Flank	Axilla
Pug	4.5	M	8	8	Flank	Axilla
Crossed spitz	1	FS	8	8	Flank	Axilla
Bullmastiff	3	M	2.1	2.1	Flank	Chin
Crossed border collie	4	FS	4.9	4.9	Flank	Abdomen
Australian shepherd	2	M	7	7	Flank	Abdomen
Crossed shepherd	2.5	FS	8.8	8.8	Flank	Abdomen
Jack Russel terrier	4.5	M	5	5	Abdomen	Abdomen
Continental bulldog	1	F	8.2	8.2	Flank	Abdomen
English cocker spaniel	1	MS	6.1	6.1	Flank	Abdomen
German shorthaired pointer	3	M	6.2	6.2	Flank	Flank
Lhasa apso	10.5	FS	5.2	5.2	Flank	Abdomen
French Bulldog	3	MS	6	6	Flank	Abdomen
Crossed German shepherd	4	MS	7	7	Flank	Axilla
Newfoundland	2	F	5.1	5.1	Flank	Abdomen
Shih-tzu	9	MS	4.5	4.5	Flank	Abdomen
Labrador retriever	3.5	M	6.8	6.8	Flank	Inner thigh
Beagle	8	FS	6.4	6.4	Not indicated	Not indicated
French bulldog	1.5	FS	5.1	5.1	Flank	Neck
Golden retriever	2	MS	6.3	6.3	Flank	Inner thigh
Jack Russel terrier	1.5	M	8.3	8.3	Abdomen	Axilla
Jack Russel terrier	5	FS	8.2	8.2	Abdomen	Abdomen
Brittany spaniel	5	MS	5	5	Flank	Inner thigh
Crossbreed	4	FS	7.5	7.5	Flank	Thorax
French bulldog	1	FS	6.2	6.2	Flank	Abdomen
West highland white terrier	4	M	9.5	9.5	Flank	Abdomen
West highland white terrier	3	M	10	10	Abdomen	Abdomen
French bulldog	5	M	7.5	7.5	Abdomen	Inner thigh
Teckel	2.5	MS	9.2	9.2	Not indicated	Not indicated
Yorkshire terrier	3	FS	7.8	7.8	Axilla	Abdomen
Shih-tzu	5.5	FS	7.5	7.5	Thigh	Neck
Crossed boxer	4	F	8.4	8.4	Back	Neck

Abbreviations: CADESI-04, Canine Atopic Dermatitis Extent and Severity Index 4; F, female; FS, female spayed; M, male; MS, male neutered; PVAS, Pruritus Visual Analog Scale.

The expression of keratinocyte differentiation markers Flg, corneodesmosin (Cdsn), and Cldn1 was evaluated using indirect immunofluorescence (IIF), and staining intensity was quantified (Figure 1). Staining patterns in healthy canine skin were identical to those reported previously (Pin et al, 2019; Roussel et al, 2015). Immunostaining with the anti-Flg antibody was severely reduced in both the nonlesional and lesional atopic skin (*P* < .0001). Cdsn staining intensity was weaker in the nonlesional atopic skin than in the healthy group (*P* = .003). A similar tendency was observed in the lesional atopic skin. Although Cldn1 also was detected in all samples, the staining was less intense in the 2 atopic groups than in the healthy group (*P* < .001).

### Morphogenesis and the development of the barrier during progressive in vitro 3-dimensional reconstruction of canine epidermis at the air–liquid interface

**Figure 2:**
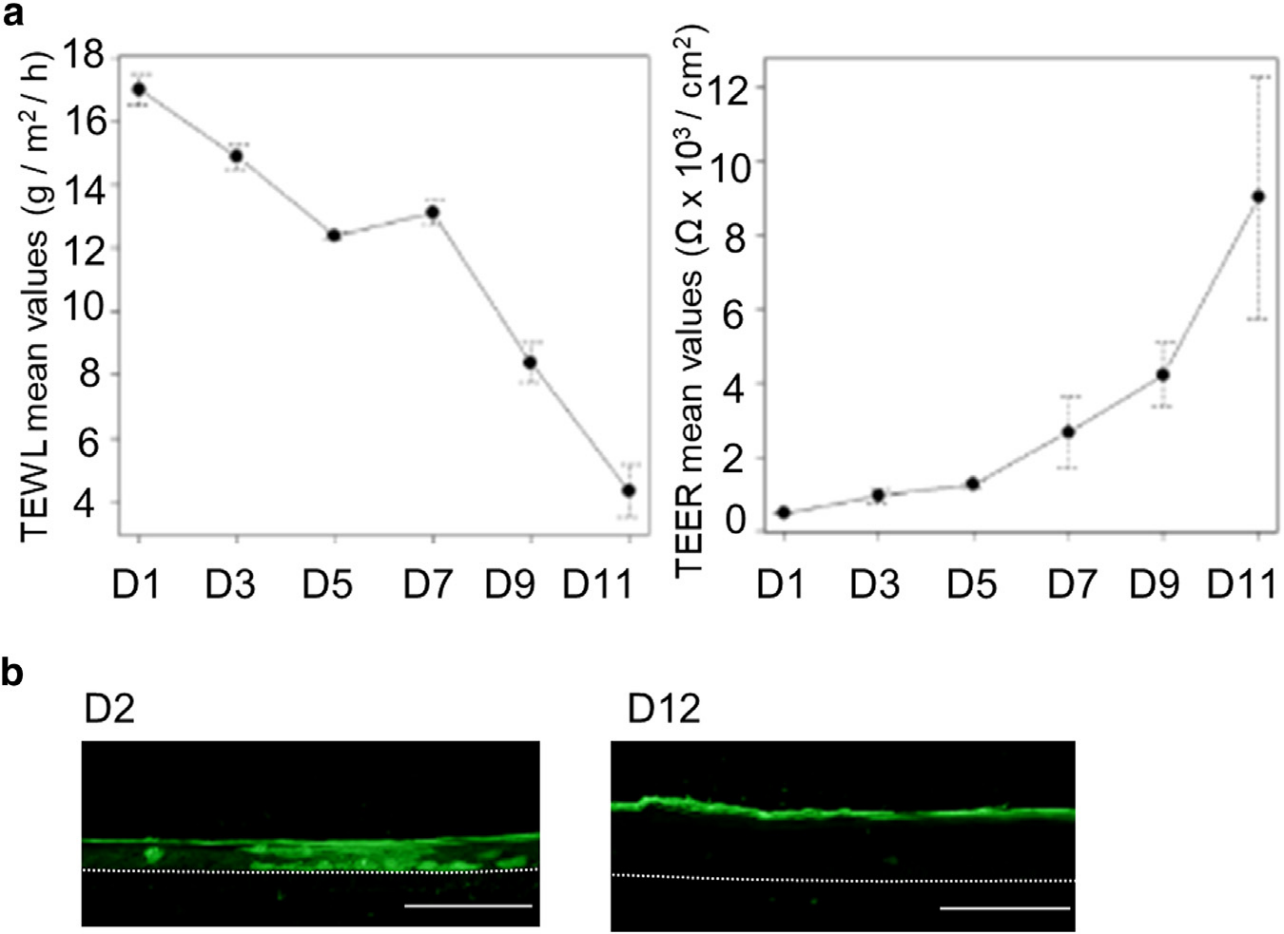
**Functional analysis of the 3D RCE barrier.** (**a**) Temporal changes in TEWL and TEER values during reconstruction from D1 to D11. n = 2. The dot represents the average value. (**b**) Penetration of Lucifer yellow dye was tested on D2 and D12. Lucifer yellow penetration assays confirmed that RCEs were functionally competent on D12, when the green dye was limited to the SC, whereas on D2, it stained the entire epidermis. Bar = 100 μm. The dotted line indicates the junction between the epithelium and the polycarbonate membrane. 3D, 3-dimensional; D1, day 1; D11, day 11; D12, day 12; D2, day 2; RCE, reconstructed canine epidermis; SC, stratum corneum; TEER, transepithelial electrical resistance; TEWL, transepidermal water loss.

**Figure 3:**
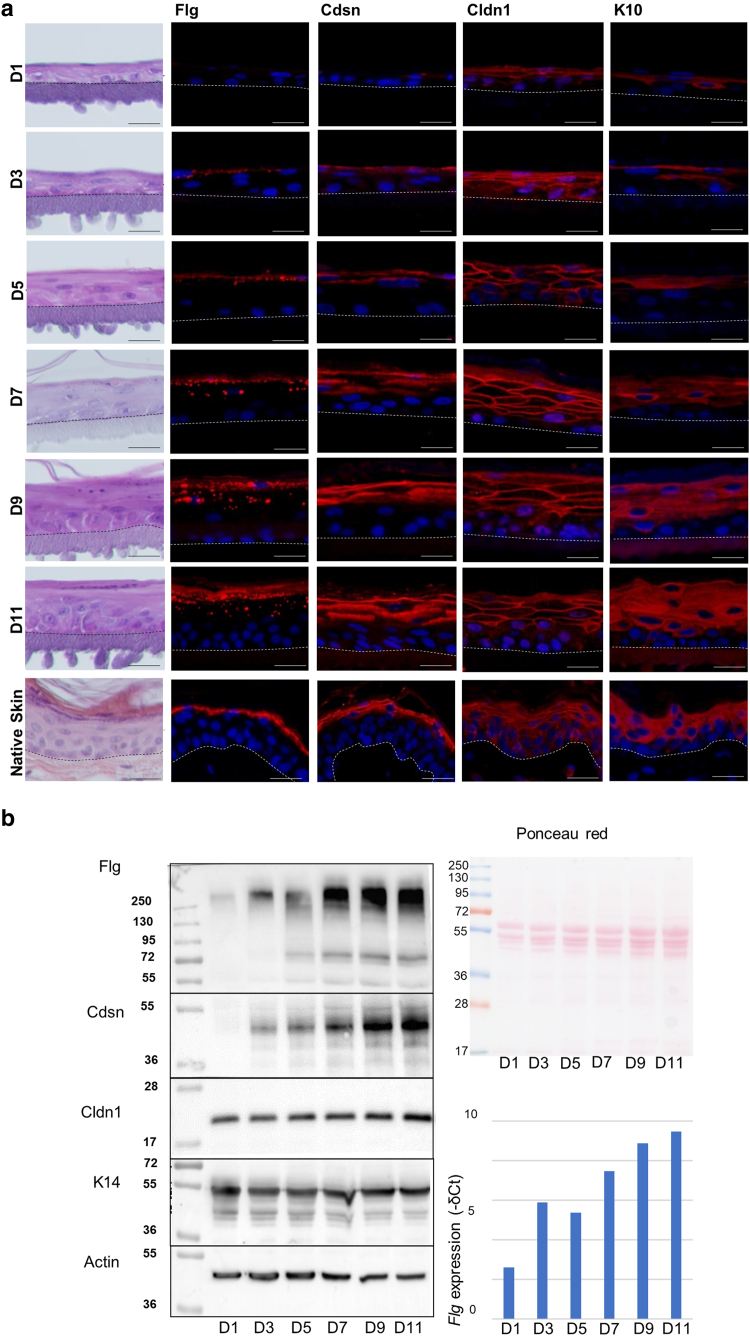
**Characterization of the RCE model.** (**a**) Images showing the development of the RCE from D1 after exposure to the air–liquid interface to D11, compared with dog-native skin. H&E-stained samples are shown in the first column on the left, followed by their corresponding immunostaining with anti-Flg, Cdsn, -Cldn1, and -K10. Bar = 25 μm. (**b**) Western blot analysis of the expression of epidermal proteins during progressive in vitro development of RCE. Total proteins were extracted from D1 to D11, separated by SDS-PAGE, transferred to nitrocellulose, stained with Ponceau red, and immunodetected with antibodies directed to the indicated proteins. Molecular mass markers are indicated in kDa on the left. RNA expression quantified by RT-qPCR is also shown. n = 2. D1, day 1; D11, day 11; K10, keratin 10; RCE, reconstructed canine epidermis.

**Figure 4:**
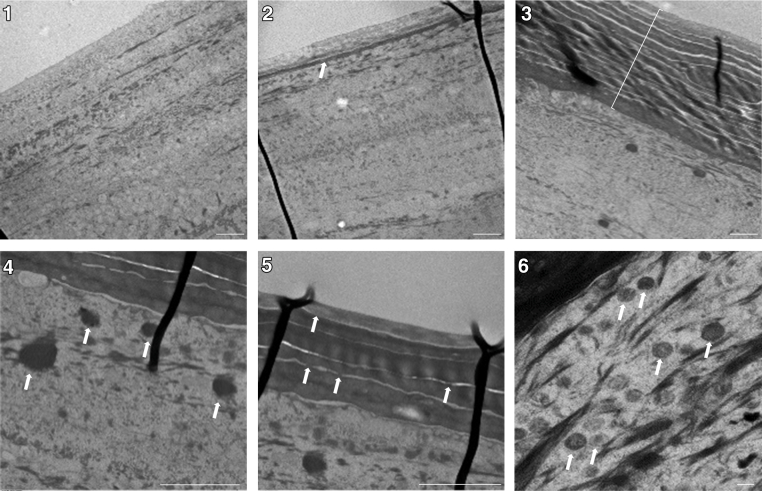
**Transmission electron microscopy images of reconstructed canine epidermis through their development.** On day D1, SC is not yet visible (1). The first corneocyte layer appeared starting on D3 (2, arrow). SC thickness increased progressively until fully developed on D11 (3, bracket). Keratohyalin granules (4, arrows) and corneodesmosomes (5, arrows) were visible from D5 on. Vesicular structures in the granular layer (probably corresponding to lamellar bodies) were visible from D3 on but become clearer in the later stages of epidermal differentiation (D9–D11) (6, arrows). Bars = 2 μm (for 1–3) and 200 nm (for 4–6). D1, day 1; D11, day 11; D3, day 3; D5, day 5; D9, day 9; SC, *stratum corneum*.

Primary keratinocytes from the skin of 2 different dogs were used to produce 3-dimensional RCE at the air–liquid interface, almost exactly as described for reconstructed human epidermis (Frankart et al, 2012). Because the canine model has not yet been described in detail in the literature (Serra et al, 2007; Yamazoe et al, 2007), we characterized the epidermis throughout the process of reconstruction in terms of the molecular composition and functionality of the barrier, from day 1 after lifting the culture at the air–liquid interface until day 11. Transepidermal water loss (TEWL) decreased progressively from day 1 (17 g/m^2^/h on average) to day 11 (4.3 g/m^2^/h). Transepithelial electrical resistance (TEER) evolved inversely (Figure 2), that is, it was low on day 1 (500 [range = 440–550] Ω × cm^2^) and increased until day 11 (9000 [6000–12,090] Ω × cm^2^). A progressive increase in thickness was observed in H&E-stained samples, from 1–2 keratinocyte layers on day 1 to a completely formed epidermal equivalent on day 11 (Figure 3a). From day 5 on, a very thin *stratum corneum* (SC) was observed in some areas, after which SC thickness progressively increased until day 11. Keratohyalin granules were first observed in small numbers on day 5; subsequently, they became more noticeable and were dispersed throughout the cytoplasm of granular keratinocytes. When analyzed using IIF (Figure 3a), a weak granular cytoplasmic expression of Flg appeared in the upper RCE from day 3 on. The granular pattern became progressively more apparent, and diffuse staining was observed in the lower SC. On day 11, the Flg-detection pattern was similar to the immunostaining observed in normal native epidermis. The anti-Cdsn antibody showed cytoplasmic staining of keratinocytes in the lower *stratum granulosum* and pericellular staining in the upper *stratum granulosum*. This staining was absent on day 1 and developed progressively starting on day 3. On day 11, it was similar to the immunodetection pattern of the native epidermis. The Cldn1-staining pattern was pericellular and was present in all layers of the epidermis, although staining was much weaker in the *stratum basale* and lower in *stratum spinosum*. The Cldn1-staining pattern was already present on day 1 but was very weak. The anti–keratin 10 immunostaining was strictly suprabasal, appearing from day 1 to day 3 in areas with more than 1 layer of keratinocytes. The anti–keratin 14 antibody predominantly labeled the cytoplasm of basal layer keratinocytes at all time points, although some weak staining was occasionally observed in the lower suprabasal keratinocytes. The corresponding proteins were immunodetected using western blots (Figure 3b). The anti-Flg antibody reacted intensely with a very high-molecular mass protein (>250 kDa) corresponding to the Flg precursor (pro-Flg). Two proteins of around 55 and 72 kDa were immunodetected, probably corresponding to Flg monomers. A very weak staining was visible at the pro-Flg level on day 1 whose intensity increased progressively until day 11. The anti-Cdsn antibody mainly reacted with a protein with a molecular mass of around 48 kDa that corresponds to the entire dog Cdsn. No staining was visible on day 1 but appeared weakly on day 3, after which the intensity increased progressively. The anti-Cldn1 antibody reacted with a protein with the expected molecular mass of 23 kDa; the intensity of the immunostaining generally remained constant throughout epidermal development. There were no significant changes in the amount of immunodetected keratin 14 at 55 kDa. The expression of the proteins at the mRNA level was quantified using RT-qPCR and was consistent with the results mentioned earlier (Figure 3b). When analyzed using transmission electron microscopy (TEM) (Figure 4), the appearance of the first corneocyte layer was observed on day 3. The number of corneocyte layers then increased progressively until a fully developed SC was observed on day 11. Keratohyalin granules and corneodesmosomes were visible from day 5 (Figure 3c). Vesicular structures in the granular layer, probably corresponding to lamellar bodies, were visible in the later stages of epidermal differentiation, between day 9 and day 11. Lucifer yellow penetration assays confirmed that the RCEs were functionally competent on day 12, when the green dye was limited to the SC, whereas on day 2, it stained the entire epidermis (Figure 2b). Taken together, these data highlight the similarity between the RCE and the interfollicular epidermis.

### RCE produced using keratinocytes from dogs with atopic dermatis did not reproduce the epidermal barrier defects observed in vivo

**Figure 5:**
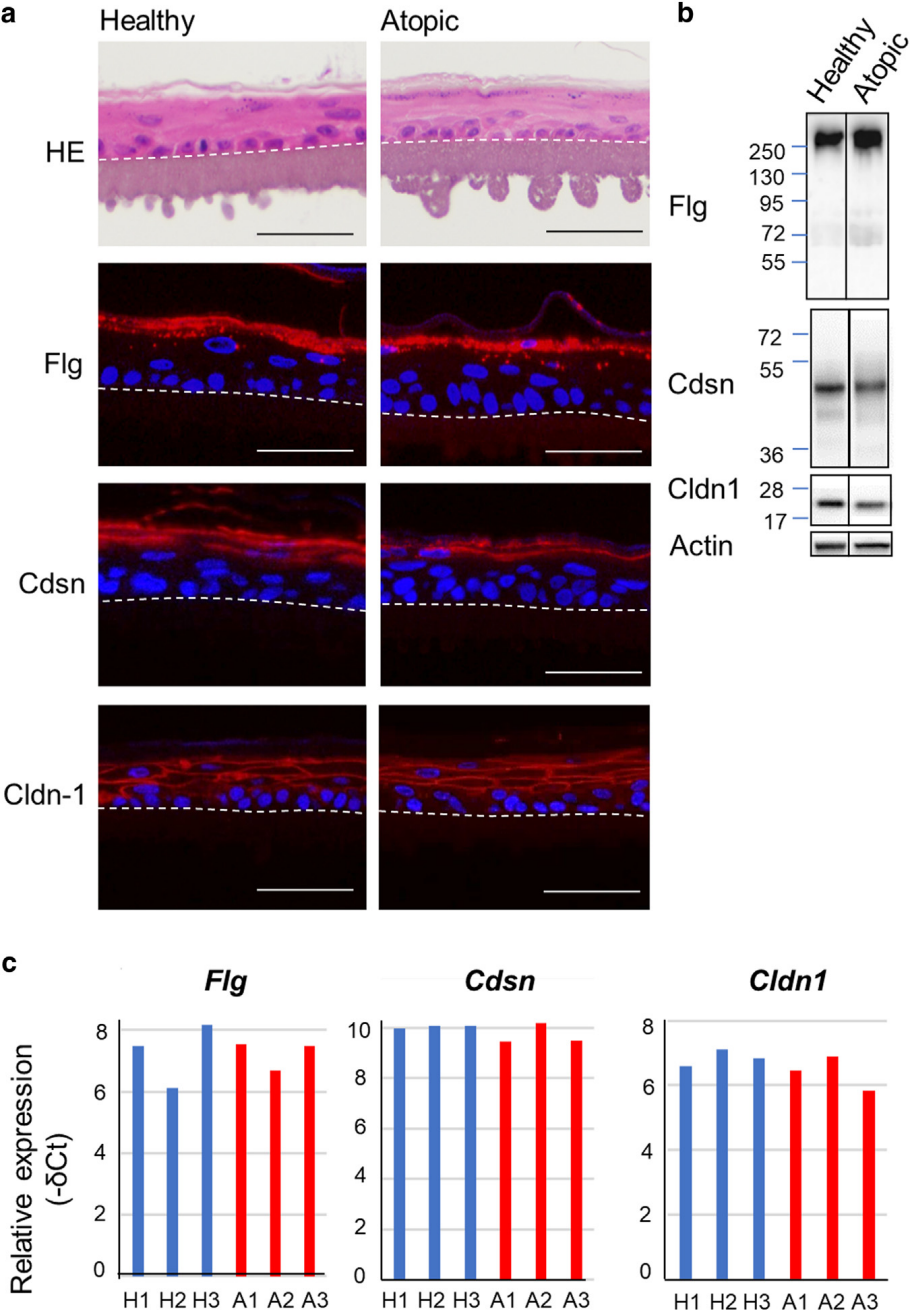
**Similar reconstructed epidermis produced with keratinocytes from either healthy or dogs with atopic dermatitis on D11.** (**a**) RCE sections were stained with H&E, and protein expression was analyzed using indirect immunofluorescence. RCEs were collected on D11. Nuclei were stained with DAPI. The images shown are representative. n = 3. Bars = 50 μm. (**b**) Western blot analysis. Molecular mass markers are indicated in kDa on the left. (**c**) Relative mRNA expression quantified by RT-qPCR from extracts of 3 healthy (H1–H3) and 3 atopic (A1–A3) RCEs. D11, day 11; RCE, reconstructed canine epidermis.

The functional, morphological, and protein expression levels of RCE produced from 3 healthy dogs (healthy RCE) and 3 dogs suffering from AD (atopic RCE) were collected at day 11 and compared. With an average value of 8.48 g/m^2^/h (range = 7.07–9.36) for the healthy RCE and 7.96 g/m^2^/h (7.60–8.60) for the atopic RCE, no statistically significant difference in TEWL values was found between the 2 groups (*P* = .52). Average TEER values were higher in the atopic than in the healthy RCE (9130 [range = 2300–12,100] vs 3251 [1925–5260] Ω × cm^2^), but the difference was not statistically significant (*P* = .21). When H&E-stained samples were examined using light microscopy, no visible differences were observed in the morphology, thickness, presence of keratohyalin granules, or the appearance of the SC between the healthy and atopic groups (Figure 5a). The protein expression of the differentiation markers Flg, Cdsn, and Cldn1 was similar in all groups at the protein and mRNA levels (Figure 5a–c).

### AD-associated epidermal barrier defects were reproduced in RCE treated with inflammatory cytokines

**Figure 6:**
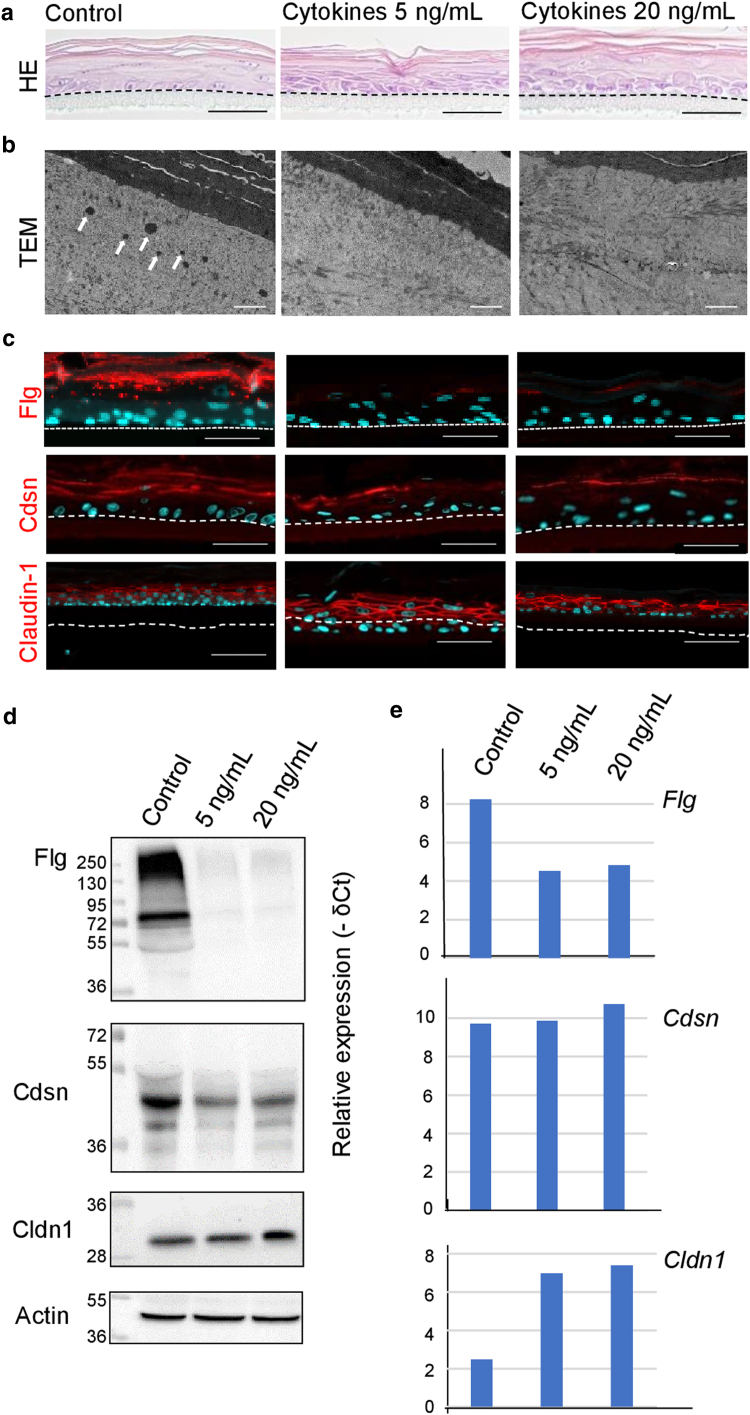
**Reduced expression of epidermal barrier proteins in reconstructed canine epidermis after challenge with cytokines.** Reconstructed canine epidermis was produced with keratinocytes from healthy dogs in the absence (control conditions) or in the presence of a cocktail of inflammatory cytokines (TNFα, IL-4, IL-13, and IL-31) at 5 or 20 ng/ml. Epidermis was collected on D11. n = 3. (**a**) Representative images of H&E-stained samples. (**b**) Transmission electron microscopy images of the same samples. The arrows point to keratohyalin granules (present only in control conditions but absent when the culture media was supplemented with cytokines). (**c**) Expression of Flg, Cdsn, and Cldn1 analyzed using indirect immunofluorescence. Bars = 50 μm (**a** and **c**) and 2 μm (**b**). (**d**) Total protein extracts were analyzed by western blot. Molecular mass markers are indicated in kDa on the left. (**e**) Gene expression, corresponding to the same experience shown in **a–d**, was analyzed by RT-qPCR and is shown as – δCt on the y axis. D11, day 11.

Because the atopic RCE did not reproduce the alterations in protein expression observed in atopic skin, we next investigated the effects of adding inflammatory cytokines to the culture medium. Healthy RCEs produced from 2 dog keratinocytes were treated with a cocktail of recombinant canine cytokines (TNFα, IL-4, IL-13, and IL-31) at 2 different concentrations (5 and 20 ng/ml) at 2-day intervals from day 6 to harvest on day 11. A decreasing trend in the TEER values was observed, with 10,700 (range = 5000–13,600) Ω × cm^2^ in the control group; 9433 (4800–11,800) Ω × cm^2^ for the group treated with the medium concentration of cytokines; and 6900 (4200–11,700) Ω × cm^2^ for the group treated with the highest concentration, but the differences were not statistically significant (*P* = .2), possibly because of high interindividual variations. The most striking feature observed upon examination of the H&E-stained samples was the almost total absence of keratohyalin granules in the RCE treated with 5 and 20 ng/ml cytokines (Figure 6a). The same feature was observed using TEM analysis (Figure 6b). Spongiosis was also observed, although not systematically, in the cytokine-treated RCE (Figure 6a). The Flg expression was reduced in a dose-dependent manner at both the protein level assessed using IIF and western blotting (Figure 6c and d) and at the mRNA level (Figure 6e). The Cdsn protein expression decreased slightly (Figure 6b), whereas Cldn1 expression remained unchanged (Figure 6c–e).

**Figure 7:**
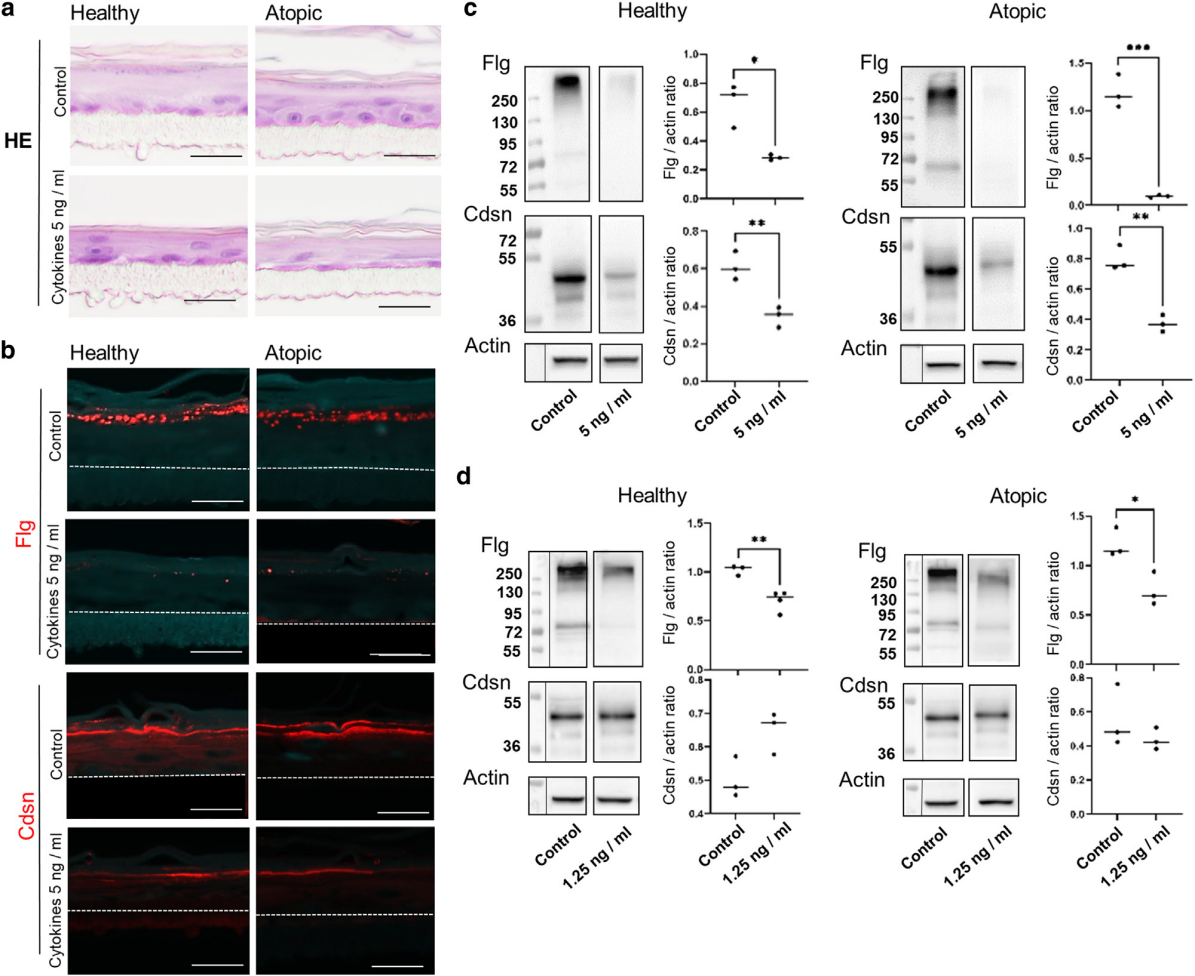
**RCEs produced with keratinocytes from healthy or dogs with atopy behave similarly when treated with a cocktail of inflammatory cytokines.** RCE was produced in the absence (control conditions) or in the presence of a cocktail of inflammatory cytokines (TNFα, IL-4, IL-13, and IL-31) at 5 or 2.5 ng/ml from D6 and collected on D11. (**a**) Representative images of H&E-stained sections of RCE. (**b**) The expression of FLG and CDSN was analyzed using indirect immunofluorescence. The dotted line indicates the junction between the epithelium and the polycarbonate membrane. Bars = 25 μm. (**c, d**) The expression of FLG and CDSN in total protein extracts was investigated using western blot, with quantification of intensity of the bands and normalization to actin on the right. Molecular mass markers are indicated in kDa on the left. n = 3. Central black line represents the median. n = 3. ∗∗∗*P* < .001, ∗∗*P* < .01, and ∗*P* < .05. CDSN, corneodesmosin; D11, day 11; D6, day 6; RCE, reconstructed canine epidermis.

Next, atopic RCE was treated with 5 ng/ml of the cytokine cocktail and produced similar results: keratohyalin granules were almost totally absent, there was a drastic reduction in Flg expression, Cdsn expression was reduced (Figure 7a–c), whereas no effect on Cldn1 expression was observed (data not shown). Finally, when healthy and atopic RCEs were treated with a lower concentration of cytokine (1.25 ng/ml) to test whether the latter potentially had a higher sensitivity of the latter, no major differences were observed (Figure 7d).

### When RCEs were treated with cytokines individually, TNFα and IL-13 had the strongest impact on Flg expression

**Figure 8:**
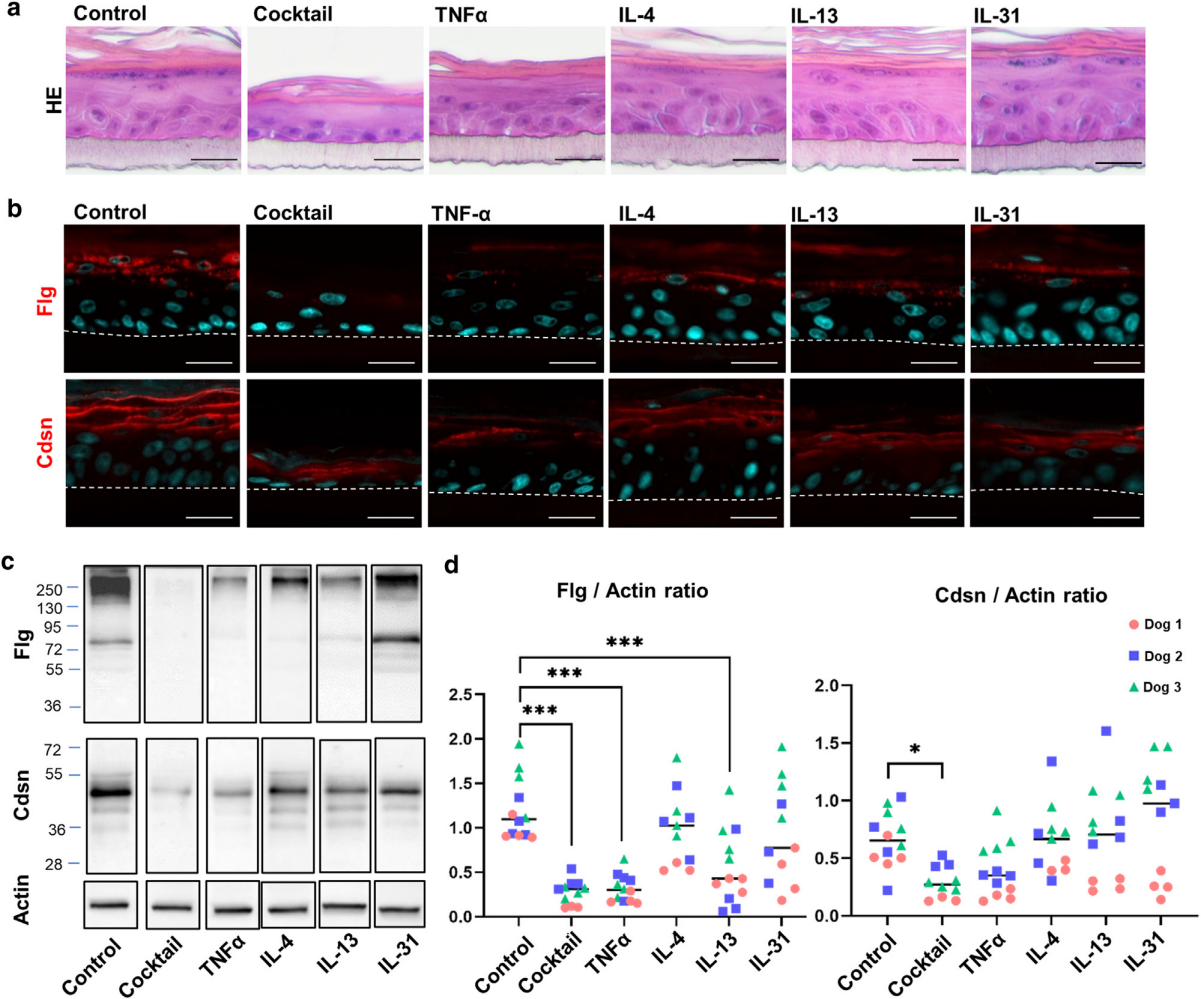
**When RCEs are treated with cytokines individually, TNFα and IL-13 are the most effective in reducing FLG expression.** (**a**) H&E staining. (**b**) Indirect immunofluorescence detection of Flg and Cdsn. Bars = 25 μm. (**c**) Western blot analysis. (**d**) Protein quantification normalized to actin. RCEs were produced with keratinocytes from 3 different dogs, as indicated by different colors (n = 3), and collected on D11. Each point corresponds to a different RCE. n = 3 The central line represents the median. ∗∗∗*P* < .001 and ∗*P* < .05. D11, day 11; RCE, reconstructed canine epidermis.

We performed individual investigations of the effects of cytokines on epidermal barrier protein expression. RCEs were produced using keratinocytes from 3 different healthy dogs. Starting from day 6, instead of a cocktail of cytokines, the RCEs were exposed to individual cytokines, each at a dose of 5 ng/ml (Figure 8), and were collected on day 11. Whereas the thickness of the epidermis was reduced when the RCEs were treated with the cytokine cocktail, it was not reduced when cytokines were used separately. Instead, the thickness resembled that observed under the control conditions. There was an overall decrease in the density of keratohyalin granules, especially with TNFα and IL-13. Flg expression was reduced by each individual cytokine, TNFα and IL-13 being the most effective ones. The effect of IL-31 on Flg expression varied depending on the keratinocyte donor. Cdsn expression was also reduced with the individual cytokines, but the difference did not reach statistical significance. Overall, no major alterations in Cldn1 expression were observed.

## Discussion

In this study, the expression of key proteins for the epidermal barrier were compared in skin samples from 32 healthy dogs and 32 atopic dogs (nonlesional and lesional samples). To the best of our knowledge, this is the largest skin sample studied in veterinary medicine with regard to AD and the skin barrier. The most striking difference observed was the marked reduction in Flg expression in the skin of dogs with atopy, in both nonlesional and lesional skin. Detection of Cdsn and Cldn1 was also reduced but to a lesser extent. In line with our results, a previously published study reported the absence of Flg immunodetection in the nonlesional skin of 4 of 18 dogs with AD (Chervet et al, 2010). The expression of Flg2, a protein closely related to Flg, has been found to be reduced in nonlesional atopic skin (Santoro et al, 2013). Reduced expression of Cdsn was observed in inflamed skin after a skin challenge with a patch test in a model of dogs with atopy sensitized to house dust mites (Olivry and Dunston, 2015). Finally, the reduced expression of Cldn1 observed in atopic skin is also in agreement with the results of 2 other studies (Olivry and Dunston, 2015; Roussel et al, 2015). Overall, the results obtained using canine skin correspond to results obtained in humans. It is well-known that in vivo expression of FLG is reduced in human atopic skin, irrespective of the presence of loss-of-function sequence variants of the *FLG* gene, secondary to skin inflammation or owing to environmental conditions (Celebi Sözener et al, 2020, Palmer et al, 2006). In dogs, to date, there has been no evidence for the existence of *Flg* sequence variants associated with AD, and this study shows how severe the influence of the inflammation on Flg expression can be. Reduced expression of Cdsn (and other corneodesmosome component proteins such as desmocolin-1 and desmoglein-1) (Lee et al, 2017) and of Cldn1 (De Benedetto et al, 2011) has also been observed in the skin of patients with AD.

The reduced expression of epidermal proteins in AD could be genetically determined or secondary to skin inflammation (Brown and McLean, 2012). To test this hypothesis, we used RCE as a model with keratinocytes from either healthy or dogs with AD. To the best of our knowledge, canine atopic keratinocytes have never been used to produce RCE. The epidermal barrier defects observed in the atopic skin in vivo, such as the drastic reduction in Flg expression, were not reproduced in RCE produced with atopic keratinocytes. We therefore hypothesized that the defects may be induced by inflammatory cytokines, such as in humans. A cocktail of canine recombinant cytokines was added to the culture medium, their range of concentration (1.25–20 ng/ml) and nature (TNFα, IL-4, IL-13, and IL-31) chosen on the basis of the literature on humans (Danso et al, 2014; Hsu et al, 2018; Rouaud-Tinguely et al, 2015; Smits et al, 2017). Th2/Th22 cytokines are overexpressed in the acute stages of canine cutaneous atopic inflammation (Olivry et al, 2016), whereas TNFα is expressed constitutively in many inflammatory conditions, including AD (Danso et al, 2014; Hsu et al, 2018; Olivry et al, 2016; Rouaud-Tinguely et al, 2015; Smits et al, 2017). The most striking effect observed when the RCE was treated with cytokines was a dramatic reduction in the expression of Flg, identical to that observed in the atopic skin. Atopic and healthy keratinocytes were found to behave in a similar way when the cytokines were added at a concentration of either 5 or 1.25 ng/ml, again suggesting that the alterations in protein expression are induced by the inflammatory milieu and are not intrinsic to the cells.

The expression of Flg was drastically reduced in both atopic skin and cytokine-treated RCE compared with that in normal skin and untreated RCE, respectively. The expression of Cdsn was also reduced in vitro in inflammatory conditions just as in atopic skin. However, the expression of Cldn1 was not altered in cytokine-treated RCE, although it was reduced in atopic skin. Further studies are required to evaluate whether these differences are due to the complexity of the inflammatory milieu observed in vivo. When human keratinocytes from individuals with atopy have been used to produce reconstructed epidermis, some differences from the control epidermis have been observed, including an increase in mitochondrial function and a heightened response to oxidative stress (Leman et al, 2022). These aspects were not however taken into consideration in this work.

When cytokines were used individually, TNFα and IL-13 significantly reduced FLG expression. All the cytokines tested in this study are known to impact human epidermal differentiation, including FLG and CDSN expression (Danso et al, 2014; Lee et al, 2017; Rouaud-Tinguely et al, 2015). IL-31 not only acts as a pruritogenic molecule in humans but also induces epidermal barrier defects (Cornelissen et al, 2012). In our study, the effect of IL-31 varied depending on the donor.

In conclusion, this study revealed a reduction in the expression of epidermal barrier proteins in the skin of dogs with atopy, the reduction in Flg being the most severe. This abnormality was not replicated in RCE produced with primary keratinocytes from either normal dogs or dogs with atopy unless inflammatory cytokines were added to the culture medium. This suggests that the skin barrier defects observed in vivo are secondary and are induced by the inflammatory milieu.

## Materials and Methods

### Animals

Client-owned dogs, aged >1 year, irrespective of breed, sex, or weight were enrolled in 2 groups defined by their history and clinical presentation. Group 1 consisted of otherwise healthy dogs that were victims of hunting (eg, boar strike) or road accidents and required surgical skin trimming. Group 2 comprised dogs with atopy undergoing an intradermal skin testing procedure. AD was diagnosed by experienced veterinary dermatologists on the basis of Favrot’s criteria (Favrot et al, 2010) and following recently published guidelines (Hensel et al, 2015). Both approval from the Science et Santé Animale n°115 Ethics Committee of the National Veterinary School of Toulouse (SSA_2020_016 and SSA_2020_019) and written owner consent were obtained in each case prior to the study.

### Canine AD diagnosis

AD was diagnosed on the basis of Favrot's criteria (Favrot et al, 2010) and following the most recent published guidelines (Hensel et al, 2015). Briefly, AD diagnosis first consisted of ruling out other causes of pruritus (ie, ectoparasite infestation [fleas, sarcoptic mange, demodicosis], bacterial pyoderma, or *Malassezia* dermatitis). Next, all the dogs underwent a hypoallergenic dietary trial lasting at least 8 weeks, followed by a challenge period to evaluate potential concurrent cutaneous adverse food reactions that could have contributed to the clinical signs. Dogs that were not adequately controlled by the hypoallergenic diet were diagnosed with AD, and the owners were advised to have their dogs undergo intradermal testing.

### Clinical evaluation

In group 1, the veterinarian in charge of admission to the veterinary clinic conducted a complete dermatological examination to ensure the absence of any other skin condition aside from the injury/injuries sustained by the dog. In group 2, on the day of enrollment, before sedation, the extent and severity of skin lesions were assessed by the investigators using the validated Canine Atopic Dermatitis Extent and Severity Index-4 (Olivry et al, 2014). The severity of pruritus was assessed by the owner using the validated Pruritus Visual Analog Scale (Hill et al, 2007; Rybnícek et al, 2009).

### Exclusion criteria

In group 1 (healthy dogs), dogs with a history of dermatological problems were not enrolled. In both groups, dogs were excluded from the study if they were receiving topical/aural and/or systemic antimicrobials, glucocorticoids (oral and injectable), ciclosporin, oclacitinib, lokivetmab, antihistamines, or essential fatty acids at enrollment or up to 3, 3–8, 6, 1, 6, 2, and 4 week(s) before inclusion, respectively.

### Skin sampling

#### Group 1

The dogs were anesthetized using the usual procedures of the veterinary care facility adapted to the condition of the dog and the severity of the injuries. Surgical skin waste was collected, and macroscopically healthy parts were preserved at +4 °C in Hanks′ Balanced Salt solution (Thermo Fisher Scientific) with penicillin–streptomycin (Sigma-Aldrich) and amphotericin B (Fungizone, Sigma-Aldrich). One 8-mm biopsy punch sample was taken and preserved in 10% neutral buffered formalin before further processing as a native control. The remaining skin was kept for the production of RCE (detailed below).

#### Group 2

Dogs were sedated for intradermal testing using butorphanol 0.3 mg/kg intravenously (Torbugesic Vet 10 mg/ml solution injectable pour chevaux, chiens et chats, Zoetis) and medetomidine 3 μg/kg intravenously (Domitor, Orion). Local anesthesia was performed with 0.5 ml of lidocaine (Laocaine, Intervet) injected subcutaneously at each biopsy site. Three 8-mm biopsies were taken, 1 from a nonlesional skin (lateral thorax) and preserved in 10% neutral buffered formalin and 2 from lesional skin. One of the latter was preserved in 10% neutral buffered formalin; the other was placed at +4 °C in Hanks′ Balanced Salt solution (Thermo Fisher Scientific) with penicillin–streptomycin (Sigma-Aldrich) and amphotericin B (Fungizone, Sigma-Aldrich) for the culture of keratinocytes.

### Histological and IIF evaluation of skin biopsies

After fixation, all the skin samples were progressively dehydrated and embedded in paraffin. A total 5-μm-thick sections were stained with H&E. Histopathological evaluation included pattern analysis and evaluation of inflammatory infiltrate and morphological changes associated with AD (Gross et al, 2005) (Table 3). Tissue sections for immunolabelling were deparaffinized, rehydrated, and rinsed with PBS. The samples were then blocked in PBS containing 3% bovine fetal serum for 1 hour, followed by overnight incubation at 4 °C with the appropriate antibodies and dilutions (Table 4). Incubation with secondary antibodies (donkey antimouse Alexa 555 or donkey antirabbit Alexa 555, Invitrogen) was performed for 1 hour at room temperature. Nuclei were stained with DAPI. The same procedure was used for the tissue sections that served as negative controls but in the absence of a primary antibody. Coverslips were mounted in Mowiol, and tissue sections were observed under a Nikon Eclipse 80i fluorescence microscope (Nikon) or an Apotome (Zeiss). To enable an objective evaluation of the IIF samples, 5 representative pictures were taken of each slide. The intensity of the immunofluorescence signal was graded using a computer imaging program (ImageJ) (Schindelin et al, 2012). The extent of the sample of epidermis concerned by primary antibody staining was traced (the same surface area appears in all the images), and mean gray values were recorded and used for statistical analysis.

**Table 3:** Scale Used for Histological Evaluation of the H&E-Stained Skin Samples

Tissue	Histological Characteristics	Quote
Epidermis	Hyperkeratosis	None (0), mild (1), moderate (2), severe (3)
	Parakeratosis	None (0), mild (1), moderate (2), severe (3)
	Acanthosis	None (0), mild (1), moderate (2), severe (3)
	Spongiosis	None (0), mild (1), moderate (2), severe (3)
	Exocytosis	None (0), mild (1), moderate (2), severe (3)
Dermis	perivascular infiltrate	None (0), mild (1), moderate (2), severe (3)
	Type of inflammatory cells	Lymphocytes: none 0, + (1), ++ (2), +++ (3)
		Histiocytic cells: none 0, + (1), ++ (2), +++ (3)
		Mast cells: none 0, + (1), ++ (2), +++ (3)
		Neutrophils: none 0, + (1), ++ (2), +++ (3)
		Eosinophils: none 0, + (1), ++ (2), +++ (3)
		Plasma cells: none 0, + (1), ++ (2), +++ (3)
Adnexa	Hyperplasia of sebaceous glands	None (0), mild (1), moderate (2), severe (3)
	Apocrine sweet glands	Dilatation? None (0), mild (1), moderate (2), severe (3)
		Hyperplastic epithelium? None (0), mild (1), moderate (2), severe (3)

**Table 4:** List of Antibodies Used in IIF and WB

Protein	Target Species	Type	Dilution IIF	Dilution WB	Reference
Flg	Human	Mouse monoclonal	1/1000	1/1000	AHF27 (Pin et al, 2019)
Cdsn	Human	Mouse monoclonal	1/1000	1/2500	G36.19 (Pin et al, 2019)
Cldn1	Human	Rabbit polyclonal	1/800	1/2500	ab15098 (Abcam) (Bizikova et al, 2011)
Keratin 14	Human	Mouse monoclonal	1/800	1/200	MA5-11599 (Thermo Fisher Scientific) (Pin et al, 2019)
Keratin 10	Mouse	Rabbit polyclonal	1/1000		PRB-159P (BioLegend) (Pin et al, 2019)
Actin	Mouse	Rabbit polyclonal		1/500	37200 (Pierce)

Abbreviations: IIF, indirect immunofluorescence; WB, western blotting.

### Production of RCE

Dog skin was incubated in 10 mg/ml of Dispase solution for 24 hours at 4 °C; the epidermis was then separated from the dermis. The epidermal fragments were treated with trypsin-EDTA solution for cell dissociation. Keratinocytes were amplified in DermaLife medium (CellSystems) in a humidified atmosphere with 5% carbon dioxide in 2-dimensional culture and stored in liquid nitrogen. For RCE, 350,000 keratinocytes in ice-cold EpiLife medium (Invitrogen Life Technologies) containing 1.5 mM calcium were seeded on polycarbonate culture inserts (surface area = 0.63 cm^2^ with pores = 0.4 mm in diameter, Merck Millipore). After 48 hours of incubation at 37 °C in a humidified atmosphere containing 5% carbon dioxide, the cells were exposed to the air–liquid interface (day 0), and 50 mg/ml vitamin C (Sigma-Aldrich) and 10 ng/ml of keratinocyte GF (Sigma-Aldrich) were added to the medium in the lower compartment. The medium was renewed at the air–liquid interface at 2-day intervals throughout the 11-day culture period. To study the progressive morphogenesis and barrier development, inserts were sampled at 2-day intervals after exposure to the air–liquid interface from day 1 until day 11. In all the other experiments, RCE was sampled on day 11.

### Cytokine treatments

Recombinant canine cytokines (IL-4 [reference AAD11563], IL-13 [reference Q9N0W9], and TNFα [reference CAA64403] from R&D Systems and IL-31 [reference C7GOW1] from CliniSciences, Nanterre, France) were added to the culture medium from day 6 and renewed at 2-day intervals until day 11.

### Analysis of RCE

#### Impact of inflammation on epidermal differentiation

For measurement of TEER and TEWL, to evaluate TEWL, a Tewameter (Tewameter TM300, Courage & Khazaka) was used, following the manufacturer’s instructions. TEER was measured using the Millicell ERS-2 (Merck KGaA). Tissues were placed in 6-well plates containing 3.5 ml of PBS and overlaid with 500 μl of PBS for the length of time required to measure electrical resistance. The 2 measurements were taken on the inserts at least 3 times and then averaged. The environmental conditions (temperature and relative humidity) were controlled during the TEWL and TEER measurement experiments.

For IIF analysis, RCE sections were immersed in 50 mM glycine buffer at pH 3.5 at 95 °C for 30 minutes, washed in distilled water before blocking in PBS containing 3% bovine fetal serum, and then processed as described for the skin biopsies.

For western blotting, each RCE was homogenized in 300 μl of Laemmli buffer, brought to a boil, and boiled for 5 minutes, 2 times. Total epidermal proteins were separated on acrylamide gels and immunodetected, first using the primary antibodies listed in Table 4 and then with peroxidase-conjugated secondary antibodies (goat antirabbit IgG-horseradish peroxidase, Southern Biotech; goat antimouse IgG-horseradish peroxidase, Bethyl Laboratories). Reaction products were detected by chemiluminescence using the ECL-prime kit (Pierce/Thermo Fisher Scientific).

#### RNA extraction and RT-qPCR

RNA was extracted using the QIAshredder (Qiagen) and RNeasy Plus Mini Kit (Qiagen). The concentration of RNA was measured using a NanoDrop 1000 Spectrophotometer (Thermo Fisher Scientific). A SuperScript III 1st strand cDNA Synthesis kit (Invitrogen, Thermo Fisher Scientific) was used to reverse transcribe RNA into cDNA. qPCR amplification was performed with the 7300 Real-Time PCR System (Thermo Fisher Scientific) using the Sybr quantitative PCR SuperMix W/ROX (Thermo Fisher Scientific). The primers used are listed in Table 5. Relative levels of gene expression among samples were determined using the Delta cycle threshold method, and the *CG14980* gene values were used for normalization.

**Table 5:** List of Primers Used in RT-qPCR

Gene	Forward Primer (5′–3′)	Reverse Primer (5′–3′)	Reference
*Flg*	GATGACCCAGACACTGCTGA	TGGTTTTGCTCTGATGCTTG	Nuttall et al (2002)
*Cdsn*	TCCCATCATCCCCAGCCATA	GTGAAACACCACCACAGGGA	
*Cldn1*	CGATGAGGTGCAGAAGATGC	GCCTGACCAAATTCATACCTG	Theerawatanasirikul et al (2012)
*K14*	AGTCCTCCAGAGATGTGACC	ACTCAAGTGTGGAGTGCTTG	Park et al (2015)
*CG14980*	GCAGGAAGGGATTCTCCAG	GGTCCAGTAAGAAATCTTCCATAA	Theerawatanasirikul et al (2012)

#### TEM

A 2-mm punch “biopsy” was performed on the culture insert on the same day as sampling. The samples were fixed in Sorensen buffer containing 2% glutaraldehyde/formaldehyde and then routinely processed for TEM. The samples were observed under a Hitachi TEM HT7700 (Japan).

#### Lucifer yellow penetration assay

To determine RCE permeability, 200 μl of a 1-mM solution of Lucifer yellow fluorescent dye (Sigma-Aldrich) was laid on the surface of the RCE for 24 hours at 37 °C before fixation and embedding in paraffin for tissue processing. Sections were analyzed using fluorescence microscopy.

### Statistical analysis

Statistical analyses were performed using R open-source software and R Commander (Fox, 2005). Normal distribution of all data was assessed using the Shapiro–Wilk normality test. If the data were normally distributed, parametric tests (Student’s *t*-test for comparison of 2 groups or ANOVA for comparison of >2 groups) were used. In these cases, Fisher’s test was performed to test the equality of variances. Welch’s correction was applied if the variances were not equal. When the data were not normally distributed, Wilcoxon rank-sum test was performed to compare 2 groups, and if >2 medians were compared, a Kruskal–Wallis test was used. A *P* < .05 was considered statistically significant. Data were presented as mean or median ± SD or data range depending on data distribution.

## Ethics Statement

Both approval from the Science et Santé Animale n°115 Ethics Committee of the National Veterinary School of Toulouse (SSA_2020_016 and SSA_2020_019) and written owner consent were obtained in each case prior to the study.

## Data Availability Statement

The data that support the findings of this study are available from the corresponding author upon reasonable request.

## ORCIDs

Daniel Combarros: http://orcid.org/0000-0003-1743-7797

Rahma Brahmi: http://orcid.org/0009-0004-1261-2060

Emma Musaefendic: http://orcid.org/0009-0000-3696-1864

Alizée Heit: http://orcid.org/0009-0005-4707-1601

Jevgenija Kondratjeva: http://orcid.org/0000-0002-2543-3895

Fabien Moog: http://orcid.org/0000-0001-8210-0264

Charline Pressanti: http://orcid.org/0000-0002-3908-7460

Line A. Lecru: http://orcid.org/0000-0001-7246-0215

Sabine Arbouille: http://orcid.org/0009-0007-5173-9357

Catherine Laffort: http://orcid.org/0009-0004-9509-3584

Dominique Goudounèche: http://orcid.org/0000-0002-0338-5079

Jessie Brun: http://orcid.org/0000-0002-5631-4653

Michel Simon: http://orcid.org/0000-0003-3655-6329

Marie-Christine Cadiergues: http://orcid.org/0000-0001-6909-0153

## Conflict of Interest

The authors state no conflict of interest.

## References

[bib1] Bizikova P., Linder K.E., Olivry T. (2011). Immunomapping of desmosomal and nondesmosomal adhesion molecules in healthy canine footpad, haired skin and buccal mucosal epithelia: comparison with canine pemphigus foliaceus serum immunoglobulin G staining patterns. Vet Dermatol.

[bib2] Brown S.J., McLean W.H.I. (2012). One remarkable molecule: filaggrin. J Invest Dermatol.

[bib3] Celebi Sözener Z., Cevhertas L., Nadeau K., Akdis M., Akdis C.A. (2020). Environmental factors in epithelial barrier dysfunction. J Allergy Clin Immunol.

[bib4] Chervet L., Galichet A., McLean W.H., Chen H., Suter M.M., Roosje P.J. (2010). Missing C-terminal filaggrin expression, NFkappaB activation and hyperproliferation identify the dog as a putative model to study epidermal dysfunction in atopic dermatitis. Exp Dermatol.

[bib5] Cornelissen C., Marquardt Y., Czaja K., Wenzel J., Frank J., Lüscher-Firzlaff J. (2012). IL-31 regulates differentiation and filaggrin expression in human organotypic skin models. J Allergy Clin Immunol.

[bib6] Danso M.O., van Drongelen V., Mulder A., van Esch J., Scott H., van Smeden J. (2014). TNF-α and Th2 cytokines induce atopic dermatitis-like features on epidermal differentiation proteins and stratum corneum lipids in human skin equivalents. J Invest Dermatol.

[bib7] De Benedetto A., Rafaels N.M., McGirt L.Y., Ivanov A.I., Georas S.N., Cheadle C. (2011). Tight junction defects in patients with atopic dermatitis. J Allergy Clin Immunol.

[bib8] Favrot C., Steffan J., Seewald W., Picco F. (2010). A prospective study on the clinical features of chronic canine atopic dermatitis and its diagnosis. Vet Dermatol.

[bib9] Fox J. (2005). The R commander: a basic-statistics graphical user interface to R. J Stat Softw.

[bib10] Frankart A., Malaisse J., De Vuyst E., Minner F., de Rouvroit C.L., Poumay Y. (2012). Epidermal morphogenesis during progressive in vitro 3D reconstruction at the air-liquid interface. Exp Dermatol.

[bib11] Freudenberg J.M., Olivry T., Mayhew D.N., Rubenstein D.S., Rajpal D.K. (2019). The comparison of skin transcriptomes confirms canine atopic dermatitis is a natural homologue to the human disease. J Invest Dermatol.

[bib12] Gross T.L., Ihrke P.J., Walder E.J., Affolter V.K. (2005).

[bib13] Hensel P., Santoro D., Favrot C., Hill P., Griffin C. (2015). Canine atopic dermatitis: detailed guidelines for diagnosis and allergen identification. BMC Vet Res.

[bib14] Hensel P., Saridomichelakis M., Eisenschenk M., Tamamoto-Mochizuki C., Pucheu-Haston C., Santoro D. (2024). Update on the role of genetic factors, environmental factors and allergens in canine atopic dermatitis. Vet Dermatol.

[bib15] Hill P.B., Lau P., Rybnicek J. (2007). Development of an owner-assessed scale to measure the severity of pruritus in dogs. Vet Dermatol.

[bib16] Hsu C.Y., Lecland N., Pendaries V., Viodé C., Redoulès D., Paul C. (2018). Stabilization of microtubules restores barrier function after cytokine-induced defects in reconstructed human epidermis. J Dermatol Sci.

[bib17] Huet F., Severino-Freire M., Chéret J., Gouin O., Praneuf J., Pierre O. (2018). Reconstructed human epidermis for in vitro studies on atopic dermatitis: a review. J Dermatol Sci.

[bib18] Lee U.H., Kim B.E., Kim D.J., Cho Y.G., Ye Y.M., Leung D.Y. (2017). Atopic dermatitis is associated with reduced corneodesmosin expression: role of cytokine modulation and effects on viral penetration. Br J Dermatol.

[bib19] Leman G., Pavel P., Hermann M., Crumrine D., Elias P.M., Minzaghi D. (2022). Mitochondrial activity is upregulated in nonlesional atopic dermatitis and amenable to therapeutic intervention. J Invest Dermatol.

[bib20] Marsella R., De Benedetto A. (2017). Atopic dermatitis in animals and people: an update and comparative review. Vet Sci.

[bib21] Nakajima S., Nomura T., Common J., Kabashima K. (2019). Insights into atopic dermatitis gained from genetically defined mouse models. J Allergy Clin Immunol.

[bib22] Nuttall T.J., Knight P.A., McAleese S.M., Lamb J.R., Hill P.B. (2002). Expression of Th1, Th2 and immunosuppressive cytokine gene transcripts in canine atopic dermatitis. Clin Exp Allergy.

[bib23] Olivry T., Dunston S.M. (2015). Expression patterns of superficial epidermal adhesion molecules in an experimental dog model of acute atopic dermatitis skin lesions. Vet Dermatol.

[bib24] Olivry T., Mayhew D., Paps J.S., Linder K.E., Peredo C., Rajpal D. (2016). Early activation of Th2/Th22 inflammatory and pruritogenic pathways in acute canine atopic dermatitis skin lesions. J Invest Dermatol.

[bib25] Olivry T., Saridomichelakis M., Nuttall T., Bensignor E., Griffin C.E., Hill P.B. (2014). Validation of the Canine Atopic Dermatitis Extent and Severity Index (CADESI)-4, a simplified severity scale for assessing skin lesions of atopic dermatitis in dogs. Vet Dermatol.

[bib26] Palmer C.N.A., Irvine A.D., Terron-Kwiatkowski A., Zhao Y., Liao H., Lee S.P. (2006). Common loss-of-function variants of the epidermal barrier protein filaggrin are a major predisposing factor for atopic dermatitis. Nat Genet.

[bib27] Park W.J., Park B.J., Song Y.J., Lee D.H., Yuk S.S., Lee J.B. (2015). Analysis of cytokine production in a newly developed canine tracheal epithelial cell line infected with H3N2 canine influenza virus. Arch Virol.

[bib28] Pin D., Pendaries V., Keita Alassane S., Froment C., Amalric N., Cadiergues M.C. (2019). Refined immunochemical characterization in healthy dog skin of the epidermal cornification proteins, filaggrin, and corneodesmosin. J Histochem Cytochem.

[bib29] Rouaud-Tinguely P., Boudier D., Marchand L., Barruche V., Bordes S., Coppin H. (2015). From the morphological to the transcriptomic characterization of a compromised three-dimensional in vitro model mimicking atopic dermatitis. Br J Dermatol.

[bib30] Roussel A.J., Bruet V., Marsella R., Knol A.C., Bourdeau P.J. (2015). Tight junction proteins in the canine epidermis: a pilot study on their distribution in normal and in high IgE-producing canines. Can J Vet Res.

[bib31] Rybnícek J., Lau-Gillard P.J., Harvey R., Hill P.B. (2009). Further validation of a pruritus severity scale for use in dogs. Vet Dermatol.

[bib32] Santoro D., Marsella R., Ahrens K., Graves T.K., Bunick D. (2013). Altered mRNA and protein expression of filaggrin in the skin of a canine animal model for atopic dermatitis. Vet Dermatol.

[bib33] Schindelin J., Arganda-Carreras I., Frise E., Kaynig V., Longair M., Pietzsch T. (2012). Fiji: an open-source platform for biological-image analysis. Nat Methods.

[bib39] Serra M, Brazís P, Puigdemont A, Fondevila D, Romano V, Torre C (2007). Development and characterization of a canine skin equivalent. Exp Dermatol.

[bib34] Smits J.P.H., Niehues H., Rikken G., van Vlijmen-Willems I.M.J.J., van de Zande G.W.H.J.F., Zeeuwen P.L.J.M. (2017). Immortalized N/TERT keratinocytes as an alternative cell source in 3D human epidermal models. Sci Rep.

[bib35] Theerawatanasirikul S., Sailasuta A., Thanawongnuwech R., Suriyaphol G. (2012). Alterations of keratins, involucrin and filaggrin gene expression in canine atopic dermatitis. Res Vet Sci.

[bib36] Thyssen J.P., Kezic S. (2014). Causes of epidermal filaggrin reduction and their role in the pathogenesis of atopic dermatitis. J Allergy Clin Immunol.

[bib37] Tsakok T., Woolf R., Smith C.H., Weidinger S., Flohr C. (2019). Atopic dermatitis: the skin barrier and beyond. Br J Dermatol.

[bib38] Weidinger S., Novak N. (2016). Atopic dermatitis. Lancet.

[bib40] Yamazoe K, Miyamoto S, Hikosaka Y, Kitagawa K, Watanabe K, Sakai H (2007). Three-dimensional culture of keratinocytes and the formation of basement membrane for canine footpad substitute. J Vet Med Sci.

